# Evaluation of Google Glass Technical Limitations on Their Integration in Medical Systems

**DOI:** 10.3390/s16122142

**Published:** 2016-12-15

**Authors:** Antonio Martinez-Millana, Jose-Luis Bayo-Monton, Aroa Lizondo, Carlos Fernandez-Llatas, Vicente Traver

**Affiliations:** 1 Instituto Universitario de Investigación de Aplicaciones de las Tecnologías de la Información y de las Comunicaciones Avanzadas (ITACA), Universitat Politecnica de Valencia, Camino de Vera S/N, Valencia 46022, Spain; jobamon@itaca.upv.es (J.-L.B.-M.); arligar@itaca.upv.es (A.L.); carferll@itaca.upv.es (C.F.-L.); vtraver@itaca.upv.es (V.T.); 2 Unidad Mixta de Reingeniería de Procesos Sociosanitarios (eRPSS), Instituto de Investigación Sanitaria del Hospital Universitario y Politecnico La Fe, Bulevar Sur S/N, Valencia 46026, Spain

**Keywords:** Google Glass, throughput, integration, surgery, imaging, medical systems

## Abstract

Google Glass is a wearable sensor presented to facilitate access to information and assist while performing complex tasks. Despite the withdrawal of Google in supporting the product, today there are multiple applications and much research analyzing the potential impact of this technology in different fields of medicine. Google Glass satisfies the need of managing and having rapid access to real-time information in different health care scenarios. Among the most common applications are access to electronic medical records, display monitorizations, decision support and remote consultation in specialties ranging from ophthalmology to surgery and teaching. The device enables a user-friendly hands-free interaction with remote health information systems and broadcasting medical interventions and consultations from a first-person point of view. However, scientific evidence highlights important technical limitations in its use and integration, such as failure in connectivity, poor reception of images and automatic restart of the device. This article presents a technical study on the aforementioned limitations (specifically on the latency, reliability and performance) on two standard communication schemes in order to categorize and identify the sources of the problems. Results have allowed us to obtain a basis to define requirements for medical applications to prevent network, computational and processing failures associated with the use of Google Glass.

## 1. Introduction

Nowadays, the population pyramid is inverted [1]. The population is increasingly aging and health care systems are adapting their services to provide coverage for the wide range of needs generated by this change [2]. One of the most important improvements is the use of information and communications technology to support medical interventions and processes, a discipline named as eHealth [3]. In this context, Google Glass (Google Inc., Mountain View, CA, USA) is a device that can be very useful in medical applications, as it allows the presentation of information in a simple and ergonomic way, in real-time to the user.

Google Glass was developed in Google X [4], a facility devoted to the development of high-technology innovative products. First prototypes dated from 2011 and were very heavy, but during the following years, size and weight were dramatically reduced. The device was released to the market in 2013 for beta-testers, and one year later in 2014, opened to general public [5]. Since then, Google Glass began to receive criticism and even legal actions, mainly due to the concern of privacy issues. Because of this controversy, Google Inc. announced, in January 2015, that it would stop its production, but made a compromise to continue with product development to create an improved version [6].

Despite the temporal product retirement, the concept driven by Google Glass has created a significant impact. On the one hand, it has ignited the development of head-mounted displays such as *Microsoft Hololens* and *Oculus Rift*, and other several solutions available in market and pre-market stages. On the other hand, it has made several inroads on their application to general medicine and specific domains [7], boosting the exploitation of wearable devices in the public health care sector. A recent review [8] summarizes the key enabling technologies that will drive next generation clinical applications. The review concludes that the field of wearable technology is one of the greatest opportunities, whereas there are major concerns about the integration of several, heterogeneous types of information and communication technologies.

Nevertheless, even in the product’s short life-time, the literature has gathered many experiences of development, tests and assessment of medical solutions using Google Glass in many fields of medicine and public health. Among the most widespread applications are surgery [9,10,11], ophthalmology [12,13], cardiology [14], emergency [15] and bedside [16]. Most studies show promising results but list a number of shortcomings when using Google Glass. Major limitations highlighted by authors are the short battery life in stand-alone use, which is less than 60 min [17]. Likewise, studies of evaluation and comparison with similar technologies (e.g., Go Pro^®^ Hero) reveal limitations in the quality of the images and the inability to indicate points of interest in the images with such a low resolution, a fact that affects the image interpretation. Other concerns spotted in the studies are related to the security and privacy of data (images, texts and sounds) that Google Glass records and stores [18].

Broadcasting medical interventions currently stands as the most important medical application of Google Glass. In 2014, a colo-rectal consultant streamed a liver resection while he was addressing questions live from an audience of more than 10,000 surgery students from more than 100 countries [19]. This shows the potential to improve patient care as relevant experts can be consulted in real-time without geographical boundaries, but it is seriously affected by hardware and network limitations, because, most of the cited studies afflict technical failures in the transmission of images, communication errors (connection lost) and sudden restarts while using the device.

Such limitations are in the scope of several research fields [20,21] on the development of new communication paradigms and computational architectures to optimize key performance indicators such as the latency, the computational overload and data quality.

In the scope of our research, limitations on the medical use of Google Glass originate from technical aspects, but the extent to which these technical aspects are preventable is still unknown.

In this study, we present an analysis of the technical limitations of Google Glass. A test bench using two well-established communication schemes has been used to identify and detect the optimal characteristics that allow a proper use of Google Glass in medical systems. A major finding reveals that even though there are not significant differences in the type of protocol with respect to the device latency, some of the exceptions that cause the automatic reset could be avoided by properly managing images’ delivery according to their size.

The rest of the manuscript is organized in four main sections: Material and Methods, in which we describe the device and the experiment’s set-up, the key metrics for the performance, and the statistical methods of the analysis; Results, in which we present the major findings achieved for each key metric; Discussion, in which we interpret the obtained results and contextualize them in the current trends of medical systems development; and Conclusion, in which we highlight the main end-point of our work.

## 2. Materials and Methods

**Figure 1:**
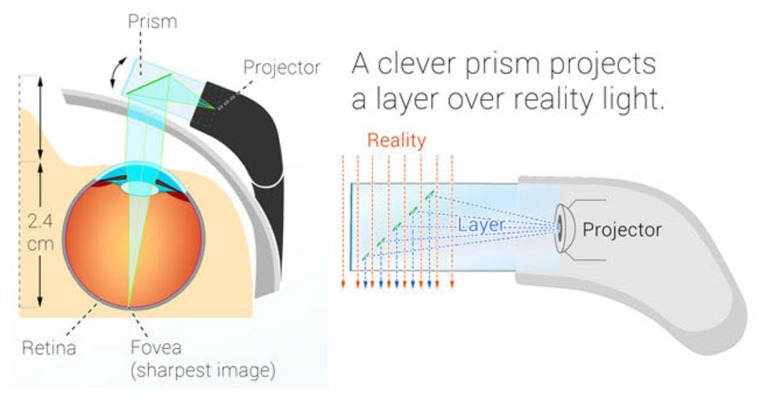
Physical schematic of Google Glass. Source: Google Glass Inc.

Google Glass is a wearable device based on a particular Android operating system. Google Glass display is equipped with a prism and a LED beamer which projects an image on the top right of the right eye using polarizing filters and semi-reflective mirrors (Figure 1). This configuration provides the user with the feeling that the image floats inside a transparent prism, without limiting the natural field of vision. This piece is adjustable to the shape of the user’s face and the distance to the eye. The device also includes a transducer which passes down vibrations to the skull near the ear to transmit sound, instead of using the auditory channel. The device has incorporated a lateral touch-pad, near to the reflection prism, to interact with the device. Moreover, it has a digital 2528 × 1856 pixels resolution camera which takes pictures in jpg format and records 720 p video in mp4 format. It is equipped with an ARMv7 Dual 1.2 Ghz Texas Instrument Processor and the available storage in the device is 12 GB. Autonomy is managed with a small battery of 570 mAh lithium-polymer, with an operating capacity of less than an hour under intensive use and three to five hours under normal use.

Google Glass features the “Jelly-Bean” Operating System which executes Android Glass Ware applications which operate very similar to “Google Now” terminal services.

The main interface of Google Glass is shown in the manner of an horizontal or vertical menu (depending on the triggered user control). The right-side external touch-pad allows one to navigate across the items in the Operative System and applications home page. The device also provides a small set of features, such as the “head wake” that allows one to wake up the device with a nod or “wink”, which also allows one to take photos with a simple wink of the eye [22]. The last type of applications in Google Glass are the “Immersions”, which are out of the time-line interface and provide background control of the user experience. Immersions are more like traditional Android applications in their use of activities, layouts and widgets. The Glass Development Kit (GDK) enables the integration of all these features to maximize the use of hardware resources in the device, such as voice control or views on a 640 × 360 pixels interface. Regarding connectivity, Google Glass has three interfaces: Wi-Fi, Bluetooth and micro-USB. For the preparation of this study, the communication channel Wi-Fi will be used, as it has been the used one in medicine according to the literature study.

To evaluate the technical performance of Google Glass, we have implemented and compared two traditional communication schemes: one based on a TCP [23] bidirectional connection and another consisting of a web service communication schema, based on REST connectors [24].

Retrofit [25], an Application Programming Interface (API), has been use to build the REST communication scheme. This schema is based on an HTTP client for Android and java, in which the *POST* method is used with the *@BODY* tag to mark the input parameters of the evaluated method. REST packets are based on the JSON messaging system, and to preserve compatibility with Glass Ware applications, we have used GSON , a Google library to manage JSON messages. For the other schema, default TCP APIs from Java framework were used to implement the TCP communication schema. In this last case, Google Glasses were provided with a private Internet Protocol (IP) direction in the network configuration.

REST is a protocol implemented on HTTP (OSI Layer 7) and TCP transport protocol (OSI Layer 4). On the one hand, REST messages are built with a higher load and thus, TCP should provide better throughput indicators, but on the other hand, as an application layer protocol, REST enables an optimal deployment of applications in Google Glass.

**Figure 2:**
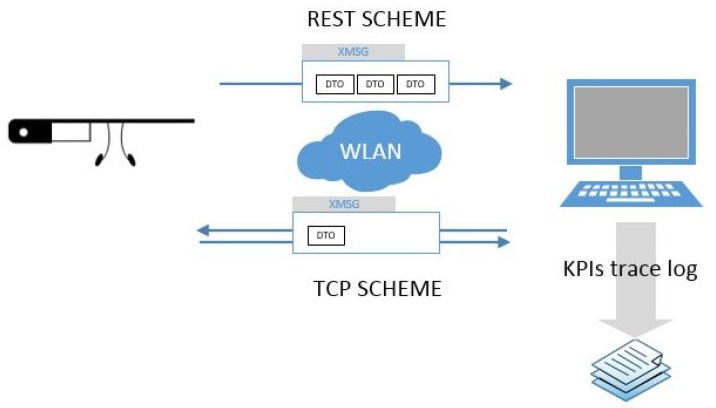
Communication schema used to perform the experiments by the implementation of TCP scheme (bottom) please define and REST (top) protocols.

Both schemes have been implemented using the *Choreographer* [26], a semantic engine capable of connecting registered services and functions (Figure 2). Using this approach, two main services for each communication schema have been defined. The *Choreographer* dispatches messages among the modules using a specific eXtensible Markup Language (XML) message protocol called eXtensible MeSsaGe (XMSG) [27]. XMSG is based on the Foundation for Intelligent Physical Agents (FIPA) recommendations [28] and Simple Object Access Protocol (SOAP) [29] headers to route and characterize messages. The XMSG protocol allows broad-/multi-cast as well as Peer to Peer (P2P) message calls using custom symbols in the destination address. Minimum transfer unit or Data Transfer Object (DTO) used was a simple object, consisting of descriptive variables and fields for message content. Performance and throughput tests were performed with a .NET framework 4.5 server and a standard 802.11 g local wireless network (WLAN) conducted with a D-LINK GO-R-Tn150 router.

The proposed comparison is based on a 10-set of systematic bench-tests using the two communication schemes with groups of 10, 100, and 1000 DTOs sent gradually, containing alpha-numeric messages, randomly generated with different sizes (0 values, 1 value, 10 and 100 values) to monitor the following Key Performance Indicators (KPI) :

- Communication Delay (Latency —milliseconds)
- Communication Success (Reliability —% success)
- Computational Load in Google Glass as regards to Memory and Central Processing Unit (CPU) use (Performance —% and Megabytes (MB)

Likewise, to assess the picture exchange and processing metrics, we have used the Early Lung Cancer Action Program (ECLAP) medical images database from Cornell University, which provides medical images in *.jpg* format of several sizes. Due to the skewed distribution of the latency parameters, a Wilcoxon signed-rank test at 95% C.I. will be used to assess the independence of intra and inter schema differences. Significance will be assumed for *p* < 0.05. Statistical and graphical analysis were done using Matlab 2016R version using Academic License.

## 3. Results

Overall results of the tests are satisfactory, as we have detected saturation points and therefore connection losses. Also, we have been able to quantify delay (latency) characteristics, reliability and performance of the device, which depends on the number of messages and the size of exchanged images.

For each test-bench (defined as a 10-set exchange of messages), the *Choreographer* has been used to send DTOs and track KPIs on REST and TCP schemes. For each iteration on the number of DTOs and the size of messages’ content, a bi-dimensional matrix was recorded with an experiment identifier. A Matlab script was implemented to automatically parse matrix and make statistical comparisons and charts.

Convenience of the GSON library for converting data type without additional configurations is confirmed. In the case of our experiments, we developed a custom serialize and deserialize method to convert complex objects (images) in alphanumeric sequences for encapsulating them in JSON messages. Furthermore, the efficiency of library Retrofit stands as a key factor to achieve a proper functioning of Google Glass, even when network problems occur, because it allows one to configure alternative addresses to make requests when others fail.

### 3.1. Communication Delay

**Figure 3:**
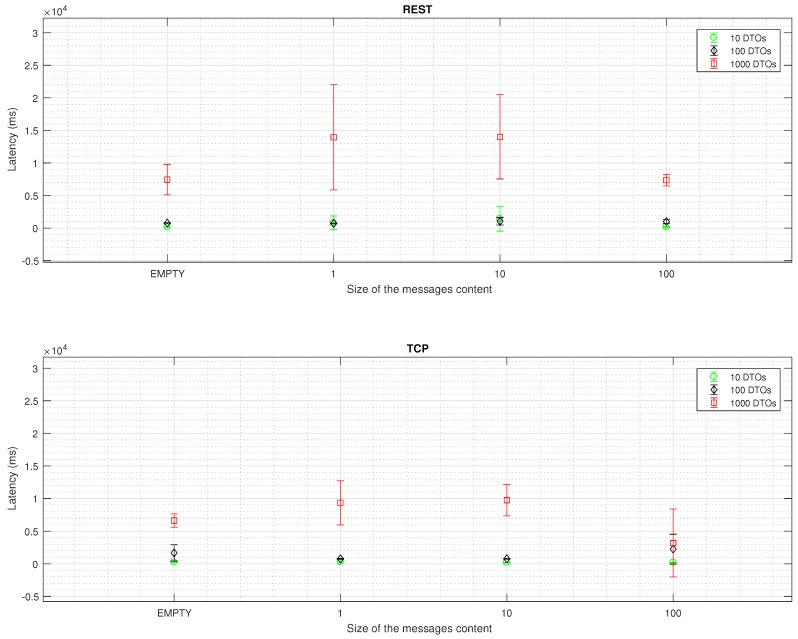
Latency in milliseconds for the REST and TCP scheme. Wilcoxon signed-rank test for a C.I. = 95% confirms statistical significant difference for each intra (*p* < 0.05) and inter scheme (*p* < 0.01) results.

Latency values for REST and TCP schemes are drawn in Figure 3. An increase in the number and size of messages is correlated to an increase in the reception time, whereas an increase in the size of the message content is correlated to a decrease. TCP schema is significantly faster than REST protocol (*p* < 0.01), as well as the distribution of the results for each bench and size (*p* < 0.05).

### 3.2. Communication Success

Regarding reliability, REST schema reaches 96.62% ± 2.35% , whereas TCP reaches 96.90% ± 1.71%. A *p* value > 0.05 (*p* = 0.882) prevents us from confirming which schema is the most reliable (Table 1).

### 3.3. Computational Load

**Figure 4:**
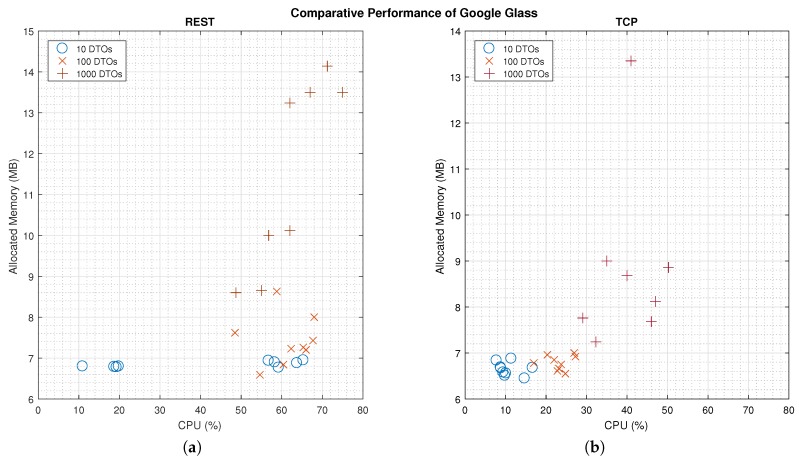
Comparison on the performance of Google Glass (CPU and Memory) for the two communication schemes. The exponential slope of the cross-points confirms our hypothesis in the Memory management and *OutOfMemory* Exception, eventhough the (**b**) figure shows a better CPU management than (**a**). (**a**) Results on the performance of REST messages management; (**b**) Results on the performance of TCP messages management.

Figure 4 shows a comparison of the computational load in the Google Glass for the two communication schemes. There are significant differences for the CPU (*p* < 0.01) and Memory (*p* < 0.05) use for each communication scheme. As expected, the TCP scheme needs less resources to process DTOs, as messages are loaded with less data. Nevertheless, the exponential shape of the results, as the amount of DTOs increases, confirms our hypothesis on the *OutOfMemory* Exception.

### 3.4. Image Processing

A total set of 14 medical images with sizes ranging from 100 KB to more than 2 MB were sent using TCP communication schema. Performance metrics such as CPU and Memory usage were tracked, as well as the delay on processing the image upon arrival. From the 64 delivered images, 46 were shown properly and the remaining 20 collapsed the device memory causing an automatic reset. To present the results, we have classified the image in three categories: Light (less than 1 MB size), Medium (between 1 and 2 MB size) and Heavy (more than 2 MB size).

**Figure 5:**
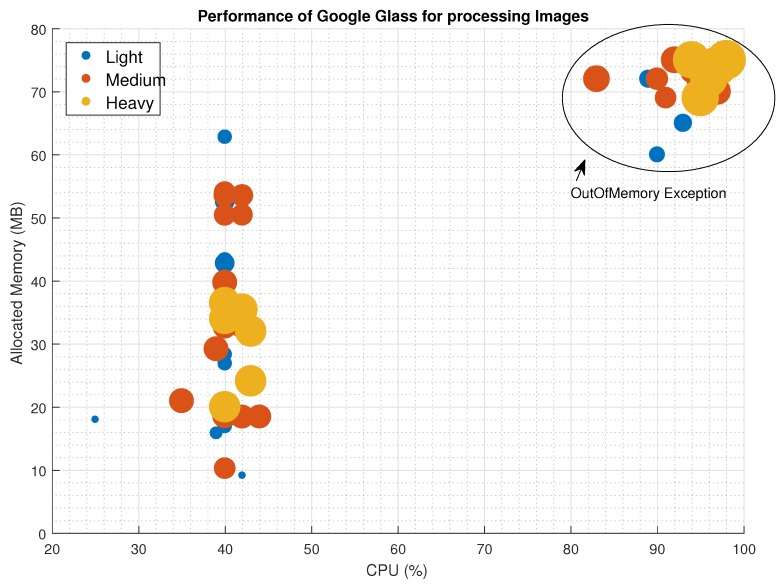
Performance (CPU and Memory use) of Google Glass for processing images of different sizes. The area of the circle is in proportion to the image size.

Figure 5 shows a scatter plot of the successful and unsuccessful delivered images and the needs of CPU and Memory. Even though there is not a clear pattern on the resources consumption related to the type of image, we can see that Light and Medium images can work in stressful conditions (40% CPU and more than 50 MB of Memory). At the same time, we can see that the image size is not linked to the device collapse; in the top-right corner we can see the images that caused the exception. When the device CPU use is over a threshold value (around 39.78%±0.35% in our experiments), the Garbage collector cannot manage the use of resources and the device performs an automatic reset.

**Figure 6:**
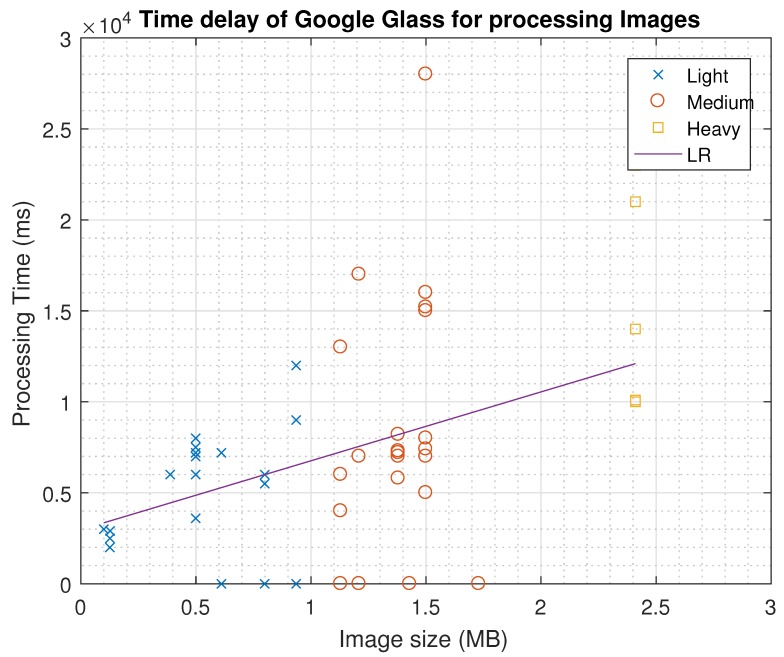
Relationship between image size and processing time of Google Glasses to show the image in the prism. Failed tests are marked with a 0 delay (overlapped with *x* axis). Linear regression (LR) goodness to fit has a $R^{2}$ = 95.1%.

With respect to the time that Google Glass needs to process the images (Delay), in Figure 6 we have noted a linear relationship among the image size and the delay. According to our experiments, as the image is serialized, prior to being sent in the TCP schema, the device performs a re-buffering operation to parse and deserialize the object, and then transform it into an image object to be projected in the prism. Approximating the distribution with a linear function in (1), ($R^{2}$ = 95.1% ), in which the image size is in MB and the time delay is in milliseconds.

(1) delay=3786×size+2975

## 4. Discussion

In this manuscript, we analyze and quantify the most widespread technical limitations of Google Glass in public health applications, as regards to previous published studies. The study has evaluated objective metrics such as latency, reliability and computational burden in sending several different data packets and different sizes of images.

We confirm that connection loss is a major issue, and results enable us to assume that the actual failure experienced in the literature happens to be an *OutOfMemory* error, since the device memory is very limited and the application closes automatically, preventing alert messages to the user before returning to the main menu. Moreover, the analysis on communication prevents us from confirming which is the suitable scheme to grant an acceptable level of success, nonetheless, results indicate that big size messages with multiple DTOs are more likely to experiment unsuccessful transmissions, as they are exposed to network fluctuations.

APIs and libraries used for communicating with Google Glass, GSON and Retrofit, have proven to be really simple and versatile when it comes to making an application to communicate with Google Glass. GSON has allowed us to customize the serialization and deserialization of data objects. Results show that Google Glass connection to the server is possible through two communication schemes: TCP and REST technology.

As for the overheads, REST messages present a higher data load than TCP, and thus, the study on the communication delay shows a significant lower latency for TCP. Nonetheless, REST messages are application-driven packets that allow less dedication on the hardware and software resources in Google Glass, to display the results to users (Figure 4). In critical applications outside the medical field, such as a remote drone controller [30], researchers have found technical limitations on the image speed processing that could be improved by optimizing network configuration. Our findings would suggest optimizing the information transfer protocol, by using REST messages to avoid critical data loss.

Nowadays, in the era of Internet of Things, REST protocols appear to be the golden choice to develop communication systems. It is clear that any technology has its advantages and disadvantages, but, according to our findings, we think that the type of application and restriction parameters should define which communication scheme accomplishes the quality requirements of service and failure tolerance.

Google Glass has been demonstrated to optimize its performance for large messages. In Figure 3, the test for 100 DTOs with the biggest size presents significantly less latency than the rest of tests. In our opinion, due to the size of the message, the device activates the garbage collector methods to re-allocate memory as the computational load in the CPU decreases.

The TCP scheme offers a better performance in terms of CPU (*p* < 0.01) and Memory (*p* < 0.05) use, but the results show an exponential slope in the use of these resources as the number of DTOs increases. Therefore, even though we would recommend a TCP scheme, it is mandatory to manage the garbage collection in the device’s memory before sending packets.

In these tests, focusing on the Google Glass behavior to send/receive messages with multiple sizes and multiple DTOs, we have shown the limitations in the processing capacity of Google Glass. The principal limitation is the amount of memory that applications can use. In addition, we have also measured the delay of the connection between server and device sending empty messages. We should mention that all tests have been conducted in a Wireless Local Area Network specifically devoted to our experiments, and thus results in the tests are not affected by external network load. However, we can conclude that the size of the data sent to the device is a crucial factor that needs to be monitored to help medical applications developers to overcome the technical limitations of Google Glass.

As expected, image processing is a major issue that does not depend on the image size, at least for the CPU and Memory management. Literature shortcomings are linked to a weak management of local memory resources, as we cannot confirm that the size of the image is linked to an *OutOfMemory* exception. Nevertheless, the time needed to process images has a linear relationship with the image size, and so, we have obtained an Equation (1) that may help researchers to identify the minimum time gap in-between the image delivery to avoid crashes.

Google Glass is a versatile and innovative wearable that can be used to improve, enhance or even create new services and tools for medical applications and systems. Positive user acceptance of the device has been evaluated and confirmed in several studies involving specialists, trainees and clinicians [18,31,32]. Acceptability of the image quality is an issue that may not be improved until the next device version is released, as the compromise between image resolution and Field of View (FoV) blocking has an unknown balance point. Some authors [33,34] in the field of image processing, have pointed out the problems regarding the visualization of tiny signals or the identification of ’Points of Interest’, however they accept the way in which the images are displayed, without distractions or FoV blocking. Nonetheless, in ophthalmology applications for patients with an impaired FoV or visual handicaps, the recording camera is a good feature to provide sound stimuli to enable them to carry on with autonomy in short displacements. However, at last, the performance of these medical applications depends on how they use the hardware, software and network features of the device, and how their limitations may affect the life cycle of sessions or communications.

Mobile and wearable technologies are progressively penetrating into specific health care applications [8]. One of the most promising fields for the use of Google Glass and other head-mounted displays is medical training [35], in which the device is used to record residents during practical lessons and on-field training, providing new perspectives for the analysis and evaluation of their interpersonal communication and manual skills without the feeling of being observed. Another successful field is its use as real-time decision support systems on patient consultations and documentation [36], by integrating electronic health records and advanced information regarding drugs/interventions into the daily communication workflow. Google Glass enables the automatic recording of sounds and images at the same time as the user interacts with the lateral touch-pad and voice commands, so physicians can double check procedures, revise check-lists, access patient data in real-time and run remote decision support systems. Last but not least, surgery and explorations stand as the flagship field for exploiting Google Glass and similar wearable devices, as it allows high quality first-person point of view image broadcasting whilst the user simultaneously accesses further information and interacts in real-time with other doctors and surgeons without the need to modify the FoV (which can be the patient him/her-self or the surgery area).

Nevertheless, as we have analyzed in this article, Google Glass has critical shortcomings that if not prevented may lead to a loss of functions in health care systems. There are a number of concerns related to the overall use of software and hardware resources, in particular to the processing performance of images and delay in the communications. For the interpretation of real-time continuous signal (i.e., ECG) or remote teleconferences, in which the delay, reliability and processing time are critical factors, Google Glass has boundaries that are likely to be managed by a proper software design and implementation, and moreover, that we hope will be overcome in future releases.

One major issue that we have not tackled, common to other mobile technologies, is the battery life, which under non-stop use does not last more than one hour, and should be taken into consideration when planning Google Glass use in health care applications.

Security and privacy are issues also identified in the literature [18]. Apart from the management of unexpected or unauthorized image and sound recordings, the way in which the information is hashed and transmitted will be a key factor for the development and certification of secure medical applications. According to our analysis, TCP provides better outcomes than REST (using HTTP), since TCP is a transport layer which does not provide information/application layer functionality; nevertheless, from a security perspective, the implementation of HTTPS will be mandatory, and thus so will be the implementation of the REST scheme.

Therefore, Google Glass is not suitable to carry out complex and heavy applications, such as large data analysis, image processing or multi-threading. Following a recent publication in the mobile technology field [37], we recommend using Google Glass as a wearable device to track and show information, and instead using dedicated servers to host the execution of complex and heavy tasks.

Time is gold in health care and Google Glass can help health professionals to have rapid access and new paths to information, but as with every medical technology, it has to be reliable.

## 5. Conclusions

Latency, reliability and performance of Google Glass depend on the technical characteristics of the software in the device and the communication schema. It is essential to know the type of traffic that a clinical application within a medical system will support, and moreover, to know what are the system requirements to ensure an efficient and effective use of the Google Glass device. TCP permits lower latency levels than REST, and thus the CPU and Memory rates remain better for TCP. The management of data as well as medical images should be optimized to prevent the application from crashing due to memory exceptions. 

## Figures and Tables

**Table 1:** Percentage of success on communications (Reliability) for the REST scheme experiments for each bulck of Data Transfer Objects (DTOs).

Message Size	REST			TCP		
	10 DTOs	100 DTOs	1000 DTOs	10 DTOs	100 DTOs	1000 DTOs
0	100	98	95.49	98	95.75	98.34
1	100	98	96.76	98	98	93.7
10	94	98	93.85	98	97.9	94.64
100	94	98	93.85	98	97.9	94.64

## Abbreviations

The following abbreviations are used in this manuscript:

CPU	Central Processing Unit
DTO	Data Transfer Object
JSON	JavaScript Object Notation
GSON	Google library for JSON
FoV	Field of View
KPI	Key Performance Indicators
OSI	Open Systems Interconnection
REST	Representational State Transfer
TCP	Transmission Control Protocol
